# A Functional Variant Rs492554 Associated With Congenital Heart Defects Modulates *SESN2* Expression Through POU2F1

**DOI:** 10.3389/fcell.2021.668474

**Published:** 2021-06-23

**Authors:** Wenke Yang, Yi Li, Jun Bai, Tao You, Kang Yi, Dingxiong Xie, Xiaowei Zhang, Xiaodong Xie

**Affiliations:** ^1^Institute of Genetics, School of Basic Medical Sciences, Lanzhou University, Lanzhou, China; ^2^Gansu Cardiovascular Institute, People’s Hospital of Lanzhou City, Lanzhou, China; ^3^School/Hospital of Stomatology, Lanzhou University, Lanzhou, China; ^4^Department of Hematology, Gansu Provincial Key Laboratory of Hematology, Second Hospital of Lanzhou University, Lanzhou, China; ^5^Department of Cardiac Surgery, Gansu Provincial Hospital, Lanzhou, China; ^6^Genetics Medicine Center, Gansu Provincial Maternity and Child-Care Hospital, Lanzhou, China

**Keywords:** congenital heart defects, *SESN2* gene, single nucleotide polymorphisms, rs492554, high-altitude, hypoxia

## Abstract

Hypoxia exposure is responsible for the high incidence of congenital heart defects (CHDs) in high-altitude areas, which is nearly 20 times higher than that in low-altitude areas. However, the genetic factors involved are rarely reported. Sestrin2 (*SESN2*), a hypoxia stress-inducible gene, protects cardiomyocyte viability under stress; thus, *SESN2* polymorphism may be a potential risk factor for CHD. We performed an association study of the *SESN2* polymorphisms with CHD risk in two independent groups of the Han Chinese population from two different altitude areas. The allele-specific effects of lead single-nucleotide polymorphisms (SNPs) were assessed by expression quantitative trait locus, electrophoretic mobility shift, and luciferase reporter assays. The molecular mechanism of *Sesn2* action against hypoxia-induced cell injury was investigated in embryonic rat-heart-derived H9c2 cells treated with or without hypoxia-mimetic cobalt chloride. SNP rs492554 was significantly associated with reduced CHD risk in the high-altitude population, but not in the low-altitude population. The protective T allele of rs492554 was correlated with higher *SESN2* expression and showed a preferential binding affinity to POU2F1. We then identified SNP rs12406992 in strong linkage disequilibrium with rs492554 and mapped it within the binding motif of POU2F1. The T-C haplotype of rs492554-rs12406992 could increase luciferase expression, whereas POU2F1 knockdown effectively suppressed it. Mechanistically, increased *Sesn2* protects against oxidative stress and cell apoptosis and maintains cell viability and proliferation. In summary, CHD-associated SNP rs492554 acts as an allele-specific distal enhancer to modulate *SESN2* expression via interaction with POU2F1, which might provide new mechanistic insights into CHD pathogenesis.

## Introduction

Congenital heart defects (CHD), structural heart defects caused by abnormal cardiovascular development during the embryonic period, are one of the most common congenital malformations in humans and the main non-infectious cause of infant death (Schneider et al., 2004; van der Linde et al., 2011). The incidence of CHD in high-altitude areas is 20 times higher than that in low-altitude areas, mainly owing to exposure to high-altitude hypoxic environments (Chun et al., 2019; Li J. J. et al., 2019). Fetal chronic hypoxia exposure promotes cardiomyocytes to withdraw from the cell cycle and become terminally differentiated cardiomyocytes, reducing the number of cardiomyocytes and increasing cardiomyocyte apoptosis (Bae et al., 2003; Zhang, 2005; Patterson and Zhang, 2010). Although the etiology of CHD involves environmental and genetic factors and their interaction (Hinton, 2013), studies on genetic factors involved in hypoxic environment exposure have been rarely reported.

Previously, three rare missense mutations (S26L-rs561648554, S254A-rs182740317, and S285C-rs762500782) in Sestrin2 (*SESN2*), which co-segregates with CHD phenotype, were identified from exome sequencing of three sporadic, non-syndromic CHD patient-parent trios in high-altitude areas (Supplementary Table 1). *SESN2* was initially described as a hypoxia-induced gene #95 (*Hi95*) (Budanov et al., 2002, 2010). SESN2 protein has two known independent functional domains, including an N-terminal oxidoreductase domain with antioxidant activity and a C-terminal domain that inhibits the mTOR signaling pathway (Kim et al., 2015). Through these two molecular functions, SESN2 exhibits cytoprotective effects in response to aberrant cellular stress. *SESN2* exerts a cardioprotective effect against damage caused by lipopolysaccharide, doxorubicin, and aging (Hwang et al., 2018; Li R. et al., 2019; Liu et al., 2020); however, its role in heart development remains unclear.

To examine the relationship between *SESN2* polymorphism and CHD susceptibility in high-altitude environments, we conducted an independent case-control study in a high-altitude Gansu population and low-altitude Beijing population. We selected three rare missense mutations (rs561648554, rs182740317, and rs762500782), a common missense mutation rs2274848, and a tag single-nucleotide polymorphism (SNP) rs492554 located in a putative enhancer element as candidate SNPs. We also studied the possible mechanism by which rs492554 affects gene expression and protects against CHD risk.

## Materials and Methods

### Study Subjects

Genetic association analyses were performed in two independent case-control groups: 778 sporadic non-syndromic CHD patients and 811 unrelated healthy controls from the Han Chinese population. The low-altitude (28–96 m) Beijing group comprised 120 CHD patients and 130 healthy controls. The high-altitude (1085–3506 m) Gansu group comprised 658 CHD patients and 681 healthy controls. Age and gender were matched between cases and controls. X-ray examination, electrocardiography, and echocardiography were used to diagnose sporadic CHD. Controls were recruited from the same region. Overall, 98 myocardial tissue samples were collected to examine *SESN2* expression (Yang et al., 2021). Written informed consent was obtained from participants or guardians. The Human Research Ethics Committees of Lanzhou University of Basic Medical Science approved the research plan (20160204).

### SNP Selection and Genotyping

Genomic DNA extraction was performed using a Cwbio^®^ Blood Genomic DNA Mini Kit (Cwbio, Beijing, China). Linkage disequilibrium (LD) analyses were conducted using HaploView version 4.2 (Broad Institute, Cambridge, MA, United States) in Han Chinese in Beijing (CHB) and Han Chinese South (CHS) populations from the 1000 Genomes Project phase 3 genotype data (1000 Genomes Project Consortium et al., 2015). In addition to rs561648554, rs182740317, and rs762500782, SNPs with a minimum minor allele frequency of more than 0.05 were considered candidate SNPs. Among the SNPs located within the *SESN2* gene and flanking sequences, those with a D′ value greater than 90 constitute strong LD blocks. For each strong LD block, it is generally sufficient to select a representative tag SNP (Barrett et al., 2005). Two online tools, RegulomeDB (Boyle et al., 2012) and HaploReg (Ward and Kellis, 2012), were used to identify the potentially functional SNPs, and to take account of the annotation of epigenetic marks in fetal heart tissue. The SNP rs492554 with a top RegulomeDB score has been identified within a putative regulatory element. The SNP rs2274848, a common missense mutation, was not in LD with any other SNP and also used as a candidate SNP. SNP genotyping was performed using the Genesky multiplex ligation detection reaction platform described previously (Yang et al., 2019). The genotyping results of approximately 5% randomly selected samples were verified using Sanger sequencing (primers listed in Supplementary Table 2).

### Epigenetic Annotation

To explore whether there are potential epigenetic functional variants, WashU Epigenome Browser was used to visualize epigenetic markers within the upstream and downstream regions of *SESN2* gene by loading chromatin immunoprecipitation sequencing (ChIP-seq) data from the Encyclopaedia of DNA Elements (ENCODE) and Roadmap Epigenomics projects (ENCODE Project Consortium, 2012; Roadmap Epigenomics Consortium et al., 2015), including histone markers (H3K4me1, H3K27ac, H3K4me3, and H3K9ac), DNase I hypersensitivity sites, and mammalian chromatin organizing protein CTCF and ChromHMM annotations in the left and right ventricles, right atrium, and fetal heart.

### Cell Culture

Human embryonic kidney 293T (HEK293T) cells and embryonic rat-heart-derived H9c2 cells were obtained from the cell bank of the Chinese Academy of Sciences (Shanghai, China) and cultured as previously described (Li et al., 2017). Briefly, HEK293T and H9c2 cells were cultured in Dulbecco’s Modified Eagle’s medium supplemented with 10% (v/v) fetal bovine serum and 1% penicillin–streptomycin in a 5% CO2 incubator at 37°C. Cells were passaged after reaching ∼80% confluence using 0.25% trypsin-EDTA (Gibco, Brooklyn, NY, United States).

### Electrophoretic Mobility Shift Assay (EMSA), Mass Spectrometry, and Transcription Factor Motif Analysis

Nucleoprotein samples of HEK293T or H9c2 cells were extracted using a Nuclear and Cytoplasmic Protein Extraction Kit (Beyotime, Shanghai, China). For each variant, 3′ biotin-labeled forward and reverse oligonucleotides were annealed to generate labeled DNA probes. According to the electrophoretic mobility shift assay (EMSA)/Gel-Shift kit protocol (Beyotime), 100× unlabeled competitor probes were incubated at 25°C for 20 min, and then, labeled probes were added. Completed reactions were run on a 6% polyacrylamide gel with 0.5× TBE running buffer and transferred to a nylon membrane for subsequent chemiluminescence imaging. EMSA results were confirmed via at least three independent experiments. Silver staining was performed to map the shifted protein band between the nylon membrane and the gel after electrophoresis. The shifted protein bands were excised and identified by Beijing Protein Innovation using the Thermo Scientific Q Exactive mass spectrometer platform. Three available online transcription factor (TF) motif databases, JASPAR (Khan et al., 2018), HOCOMOCO (Kulakovskiy et al., 2016), and PROMO (Messeguer et al., 2002) were used to predict allele-specific binding TFs. Proteins identified in predicted TFs and using mass spectrometry were considered priority TFs and the corresponding SNP as priority SNP.

### Luciferase Expression Plasmid Constructs and Luciferase Reporter Assay

Combined with the priority TF, potential epigenetic functional annotation, and haplotype information, the priority SNP was re-analyzed to determine the potential *cis*-regulatory elements. Luciferase reporter plasmids were constructed using Public Protein/Plasmid Library Biotechnology (Nanjing, China), the detailed process for plasmid construction is illustrated in Supplementary Figure 1. The putative enhancer (212 bp) and promoter (2183 bp) fragments containing the major or minor allele were cloned into the *Kpn*I/*Xho*I sites of the pGL6-Basic vector (Beyotime), followed by Sanger sequencing. For luciferase reporter assay, 24 h before transfection, approximately 1.5 × 10^4^ HEK293T cells per well were seeded in 96-well black plates. Constructed plasmids were transfected in groups using Opti-MEM (Gibco, Waltham, MA, United States) and Lipofectamine 2000 Transfection reagent (Invitrogen, Waltham, MA, United States). To detect the role of priority TFs, specific small-interfering RNA (siRNA) was used for co-transfection. After 48 h transfection, luciferase activity was measured using a Bright-Lumi^TM^ Firefly Luciferase Assay Kit (Beyotime) on a TECAN infinite M200 Pro microplate reader (Tecan, Switzerland). Luciferase assay results were obtained from three independent experiments, each performed in triplicate.

### *In silico* Analysis

The online database of Genotype-Tissue Expression (GTEx)^1^ was used to evaluate the relationship between *SESN2* polymorphism and expression in myocardial tissues. Eight sets of mRNA microarrays, GSE13834 (Cao et al., 2008), GSE14970 (Ghorbel et al., 2010), GSE17579 (Gupta et al., 2010), GSE26125 (Bittel et al., 2011), GSE35776 (O’Brien et al., 2012), GSE45821 (Votteler et al., 2013), GSE61154 (Devalla et al., 2015), and GSE150051 (Drakhlis et al., 2021), were downloaded from the Gene Expression Omnibus (GEO) database^2^ to analyze the expression of *SESN2* and functional gene sets in normal myocardial tissues. GSEA v4.0.3 software was utilized to perform Gene Set Enrichment Analysis (GSEA) with the Hallmark (v7.2) gene sets in the Molecular Signatures Database (Subramanian et al., 2005). *P* < 0.05 and false discovery rate < 0.25 were considered differentially expressed thresholds.

### Cell Transfection and Treatment

The recombinant lentiviral vector pLV-Puro-Sesn2 with full-length rat *Sesn2* coding sequence (LV-Sesn2) or empty vector pLV-Puro (LV-Control) as negative control (Beijing Gene-Van Biology Co., Ltd.) was transfected into H9c2 cells with 8 μg/mL polybrene (H8761, Solarbio, Beijing, China). After 48 h, lentivirus-infected H9c2 cells were screened using 1.5 μg/mL puromycin for 2 weeks. *Sesn2* knockdown in H9c2 cells was performed using siRNA targeting *Sesn2* (si-Sesn2). Three specific si-Sesn2 (si-Sesn2-5; 5′-GACUACCUUAGCAGCUUCUTT-3′, si-Sesn2-7; 5′-CUCAGCGAGAUCAACAAAUTT-3′, si-Sesn2-10; 5′-CAAGCAGAGACCCAUUGAATT-3′) and negative control si-NC (5′-UUCUCCGAACGUGUCACGUTT-3′) oligonucleotides were purchased from GenePharma (Shanghai, China). H9c2 cells were transfected with si-Sesn2 or si-NC using Lipofectamine 2000 in Opti-MEM and incubated for 6 h. Then, the medium was replaced with fresh culture medium. After 48 h transfection, cells were harvested to assess mRNA and protein levels by quantitative reverse-transcription polymerase chain reaction (RT-qPCR) and western blotting.

To further assess the effect of knockdown or overexpression of *Sesn2* in embryonic rat-heart-derived H9c2 cells under hypoxia, cobalt chloride (CoCl_2_) (Sigma-Aldrich) was used to establish a hypoxia-mimetic model in H9c2 cells (Li et al., 2017).

### Cell Viability and Cell Proliferation Assays

Cell viability after treatment with different concentrations of CoCl_2_ was assessed by 3-(4,5-dimethylthiazol-2-yl)-2,5-diphenyltetrazolium bromide (MTT) and lactate dehydrogenase (LDH) leakage assays. For MTT assay, H9c2 cells were seeded in 96−well plates at 1 × 10^4^ cells/well. Following 24 h incubation, cells were treated with CoCl_2_ at various concentrations (0, 100, 200, and 400 μM) for 5 h at 37°C, and 20 μL MTT solution [5 mg/mL in phosphate-buffered saline (PBS)] was then added to each well. After another 4 h incubation at 37°C, the culture medium was removed. Dimethyl sulfoxide (150 μL) was added to each well to solubilize the formazan. Absorbance at 490 nm was measured using a TECAN microplate reader. In LDH leakage assay, LDH activity in the cell culture medium was determined using a commercial LDH assay kit (Nanjing Jiancheng Bioengineering Institute, China). It was used to verify the CoCl_2_ concentration screened via MTT assay and evaluate the effect of different *Sesn2* levels on cell viability in *Sesn2*-overexpressing, *Sesn2*-knockdown, and normal control H9c2 cells under CoCl_2_-induced pseudo-hypoxic conditions.

Cell proliferation was evaluated by bromodeoxyuridine (BrdU) incorporation assay using a BrdU cell proliferation ELISA kit (Abcam, Cambridge, United Kingdom). Briefly, 1 × 10^4^ cells of each group were incubated with 120 μL labeling medium containing 20 μL diluted 1× BrdU for 5 h at 37°C. Cells were then fixed, washed, and incubated with the anti-BrdU monoclonal antibody for 1 h at room temperature. After processing with peroxidase-labeled secondary antibody and TMB peroxidase substrate, stop solution was added and absorbance was measured at 450 nm using a TECAN microplate reader.

### Quantification of Intracellular Reactive Oxygen Species (ROS) Levels and Superoxide Dismutase (SOD) Activity

Intracellular reactive oxygen species (ROS) levels were measured using dihydroethidium (DHE) (Sigma-Aldrich) staining methods. Briefly, 1 × 10^4^ cells of each group in 96-well black plates were incubated with 50 μM DHE for 1 h. Cells were then washed with PBS thrice. Red fluorescence intensity was measured at 535 nm excitation and 610 nm emission using a fluorescent microplate reader (Tecan). Then, cells were gently washed twice with PBS and total protein of each group was extracted; fluorescence intensity was normalized with protein concentration.

Superoxide dismutase (SOD) activity was quantified using commercial SOD assay kits (Nanjing Jiancheng Bioengineering Institute) as described previously (Li et al., 2017).

### Cell Cycle Analysis

Cells from each group were harvested, washed twice with cold PBS, and fixed with 70% ethanol at 4°C overnight. After 3 min centrifugation at 1500 × *g*, cell pellets were washed with cold PBS and re-centrifuged. Subsequently, cells from each group were resuspended in 500 μL 1× staining buffer containing 10 μL 50× RNase A and 25 μL 20× propidium iodide stock solution (Beyotime) and incubated at 37°C in the dark for 30 min. Cell cycle was then analyzed using a flow cytometer (BD FACS AriaIII, NY, United States). FlowJo software (Flowjo LLC, Ashland, OR, United States) was used to analyze cell cycle distribution.

### Cell Apoptosis Assays

Cell apoptosis was analyzed by measuring caspase−3 activity using a caspase-3 activity colorimetric assay kit (Solarbio) and Annexin V-PE/7-AAD dual staining. In Annexin V-PE/7-AAD dual staining, cells were harvested and washed twice with cold PBS. After 5 min centrifugation at 1000 × *g*, cell pellets of each group were resuspended in 100 μL 1× binding buffer and incubated with 5 μL of Annexin V-PE and 5 μL of 7-AAD for 15 min at room temperature in the dark. Then, 400 μL of 1× binding buffer was added and cell apoptosis was immediately assayed by flow cytometry. All data were analyzed using FlowJo Software.

### Real-Time Quantitative PCR

Total RNA from myocardial tissues or cells was extracted using TRIzol Reagent (Life Technologies, Carlsbad, CA, United States) (Yang et al., 2021). After RNA purity and concentration detection, cDNA was obtained from RNA using a Transcriptor First Strand cDNA Synthesis Kit (Roche, Switzerland). FastStart Universal SYBR Green Master (Roche, Switzerland) and Rotor-Gene Q Real-Time PCR System (Qiagen) were used for qPCR. Relative mRNA expression was calculated by the 2^–ΔΔCt^ method with β-actin as an internal control (primers listed in Supplementary Table 2).

### Western Blotting

Western blotting was performed as described previously using antibodies listed in Supplementary Table 3 (Li et al., 2017).

### Statistical Analysis

For categorical variables, differences were calculated using chi-squared test. Multiple testing was corrected using Bonferroni correction (Santos-Cortez et al., 2018). Correlations were assessed using Pearson’s correlation coefficient. For continuous variables, data are presented as mean and standard deviation. Two-tailed unpaired Student’s *t*-test was used to evaluate differences between two groups. For unequal variances, Welch’s correction was followed. One-way analysis of variance was used to evaluate differences among three or more groups. All experimental data were obtained from at least three independent experiments. *P* < 0.05 was considered statistically significant. Statistical analyses were performed using GraphPad Prism software (GraphPad Prism Software, Inc.).

## Results

### The rs492554 T Allele Significantly Reduces CHD Risk in High-Altitude Han Chinese Population

To assess the association between *SESN2* polymorphism and CHD risk, we analyzed 778 sporadic patients and 881 controls from two different altitude provinces (cities) in China. Patient demographic and clinical characteristics are presented in Table 1. Furthermore, rs561648554, rs182740317, and rs762500782 were not detected in any tested sample and were unavailable for subsequent statistical analysis. A Bonferroni-corrected *P*-value less than 0.025 was considered statistically significant for two available SNPs. For rs2274848, no association was observed in any genetic model or stratification analysis (Supplementary Tables 4, 5). The genotypes of rs492554 were in Hardy–Weinberg equilibrium in the control subjects of the Beijing, and Gansu groups (*P* = 0.43 and 0.28, respectively). The T allele of rs492554 was associated with decreased CHD risk in the Gansu group (OR = 0.73, 95% CI = 0.59–0.92, *P* = 0.008) but not in the Beijing group (OR = 0.79, 95% CI = 0.45–1.40, *P* = 0.48). The results of the other genetic models are presented in Table 2. Stratification analysis according to CHD classification or subtypes described previously showed that rs492554 was significantly associated with complex CHD, but the associations with isolated CHD and tetralogy of Fallot (TOF) were of borderline statistical significance in the Gansu group (Table 3).

**TABLE 1:** Clinical characteristics in CHD cases and controls.

Variable	Cases		Controls		*P*
		
	No.	%	No.	%	
Beijing group	120		130		
Age, years (mean ± SD)	2.50 ± 2.15		2.78 ± 2.21		0.31
Gender (Male/Female)	60/60	50/50	65/65	50/50	1.00
CHD classification					
Isolated/Complex CHD^*a*^	57/63	47.5/52.5			
Detailed CHD phenotypes					
ASD/VSD/TOF/Others	13/44/19/44				
Gansu group	658		681		
Age, years (mean ± SD)	15.06 ± 17.53		15.71 ± 15.89		0.47
Gender (Male/Female)	338/320	51.4/48.6	374/307	54.9/45.1	0.19
CHD classification					
Isolated/Complex CHD^*a*^	421/237	64.0/36.0			
Detailed CHD phenotypes					
ASD/VSD/TOF/Others	174/247/65/172				
Combined group	778		811		
Age, years (mean ± SD)	13.14 ± 16.77		13.64 ± 15.34		0.54
Gender (Male/Female)	398/380	51.2/48.8	439/372	54.1/45.9	0.24
CHD classification					
Isolated/Complex CHD^*a*^	481/297	61.8/38.2			
Detailed CHD phenotypes					
ASD/VSD/TOF/Others	187/291/84/216				

*^*a*^Isolated CHD including ASD and VSD. Complex CHD including TOF, ASD + VSD, AVSD, TOF + ASD, ASD + PDA, VSD + PDA, ASD + PS, and VSD + PS.*

*CHD, congenital heart disease; ASD, atrial septal defect; VSD, ventricular septal defect; TOF, tetralogy of Fallot; AVSD, atrioventricular septal defect; PDA, patent ductus arteriosus; PS, pulmonary stenosis; SD, standard deviation.*

**TABLE 2:** Association of rs492554 polymorphism and CHD in two independent case-control groups.

Variable	rs492554 Genotype	Case	Control	OR (95% CI)	*P*
Beijing group	CT vs. CC	24/96	30/99	0.83 (0.45–1.50)	0.54
	TT vs. CC	0/96	1/99	0.34 (0.01–8.50)	1.00
	CT + TT vs. CC	24/96	31/99	0.80 (0.44–1.50)	0.54
Gansu group	CT vs. CC	127/521	177/493	0.68 (0.52–0.88)	**0.004**
	TT vs. CC	10/521	11/493	0.86 (0.36–2.00)	0.83
	CT + TT vs. CC	137/521	188/493	0.69 (0.54–0.89)	**0.004**

*CC genotype represented the reference group in the analyses.*

*Bold *P*-values indicate significance after Bonferroni correction.*

**TABLE 3:** Stratified analysis of rs492554 by CHD subtypes in the dominant model (CT + TT vs. CC).

	Beijing group		Gansu group	
		
	*P*	OR (95% CI)	*P*	OR (95% CI)
**CHD classification^a^**				
Isolated CHD	0.57	0.76 (0.35–1.70)	0.06	0.75 (0.57–1.00)
Complex CHD	0.72	0.83 (0.40–1.70)	**0.004**	0.58 (0.40–0.84)
**Detailed CHD phenotypes**				
ASD	0.73	0.58 (0.12–2.80)	0.15	0.73 (0.49–1.10)
VSD	0.84	0.82 (0.36–1.90)	0.15	0.77 (0.55–1.10)
TOF	0.57	0.60 (0.16–2.20)	0.08	0.53 (0.27–1.00)

*^*a*^Isolated CHD including ASD and VSD. Complex CHD including TOF, ASD + VSD, AVSD, TOF + ASD, ASD + PDA, VSD + PDA, ASD + PS, and VSD + PS.*

*Bold *P*-values indicate significance after Bonferroni correction.*

*CHD, congenital heart disease; ASD, atrial septal defect; VSD, ventricular septal defect; TOF, tetralogy of Fallot; AVSD, atrioventricular septal defect; PDA, patent ductus arteriosus; PS, pulmonary stenosis.*

### The rs492554 Protective T Allele Is Associated With High *SESN2* Expression

**FIGURE 1:**
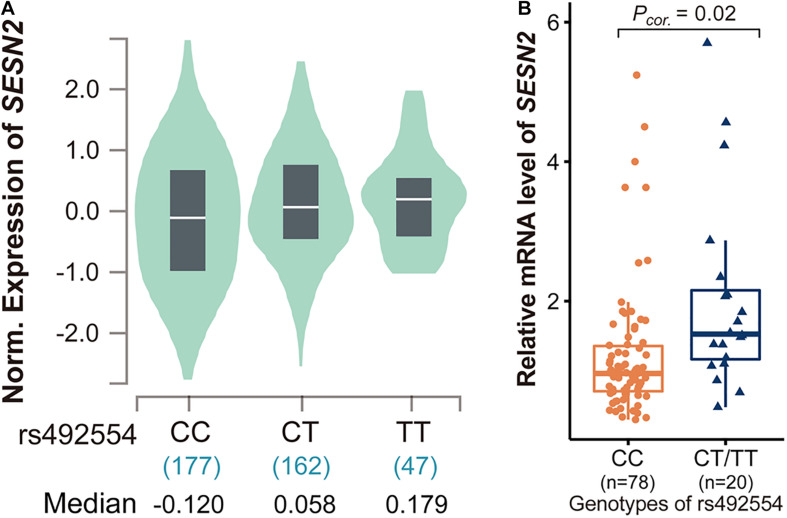
Analysis of expression quantitative trait locus (eQTL) effects at *SESN2*. **(A)** *SESN2* displayed a significant eQTL association with rs492554 genotypes (T is the protective allele and C is the risk allele) in 386 left cardiac ventricular tissues of the Genotype-Tissue Expression (GTEx) database (*P* = 2.5 × 10^–6^). **(B)** Relative mRNA expression levels of *SESN2* in 98 myocardial tissues with rs492554 genotypes. Gene expression data were analyzed by two-tailed unpaired Student *t*-test with Welch’s correction for unequal variances.

We determined whether rs492554 was associated with *SESN2* expression. Initially, the publicly available RNA-seq datasets for heart tissues from the GTEx Project (database of Genotypes and Phenotypes accession phs000424.v8.p2) revealed an association between the protective T allele of rs492554 and increased *SESN2* expression (*P* = 2.5 × 10^–6^; Figure 1A); the association was presented with a strong expression quantitative trait locus (eQTL) effect in 386 left cardiac ventricular tissues. Moreover, 98 myocardial tissues were collected to verify this association. We observed significantly higher *SESN2* expression in TT or CT carriers than in CC genotype carriers (*P* = 0.02; Figure 1B) (Supplementary Table 6). These results suggest a significant association between the T allele of rs492554 and *SESN2* upregulation.

### The rs492554 T Allele Enhances the Potential Binding Affinity of Transcription Factor POU2F1

**FIGURE 2:**
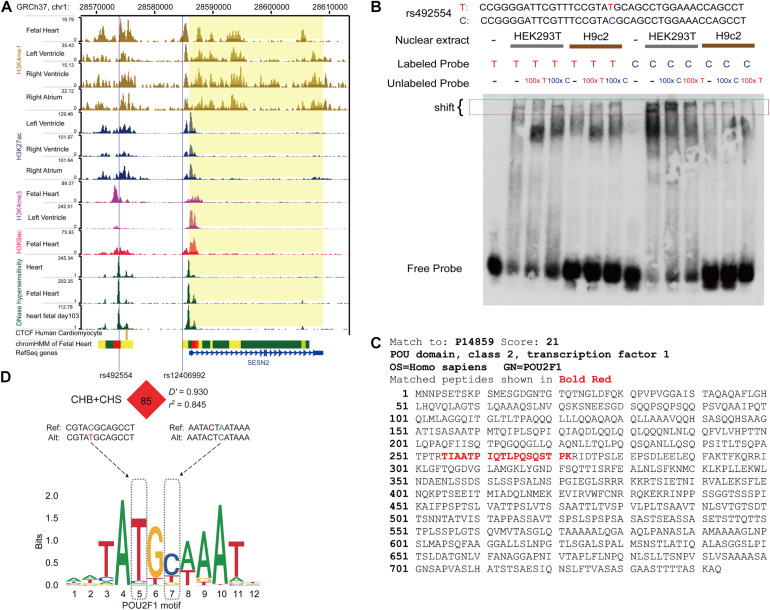
The protective allele T of rs492554 enhances the potential binding affinity of transcription factor POU2F1. **(A)** Genome browser tracks for functional annotation of the upstream and downstream flanking sequences of *SESN2* in heart or fetal heart tissues. **(B)** EMSAs were performed using biotin-labeled double-stranded oligonucleotides for the T and C alleles of rs492554 and nuclear extracts from HEK293T cells and H9c2 cells. 100× molar excess of unlabeled competitor was added in specified lanes. The rs492554 protective T allele drives preferential protein binding. Red 100× refers to the T allele-containing probe. Blue 100× refers to the C allele-containing probe. **(C)** Peptide of POU2F1 protein identified by mass spectrometry from the shifted protein bands of EMSAs. **(D)** rs12406992 is located in the putative promoter region of *SESN2* and is in strong linkage disequilibrium with rs492554 in the Han population (CHB and CHS). rs492554 and rs12406992 reside within POU2F1 DNA-binding motifs.

We then tried to identify the regulatory mechanism underlying rs492554. The ChIP-seq data from ENCODE and Roadmap Epigenomics projects were used to annotate heart- or fetal heart-specific *cis*-regulatory elements (Figure 2A). The data from the GTEx database were used to identify SNPs with an eQTL effect on *SESN2* expression. The genotype data of CHB and CHS from the 1000 Genomes Project were used to conduct imputation of SNPs in a complete linkage disequilibrium (LD; *r*^2^ = 1) with lead SNP rs492554. Therefore, the prioritized rs2481974 and rs510803 were identified as candidate functional variants with enhancer histone markers in heart or fetal heart tissues.

**FIGURE 3:**
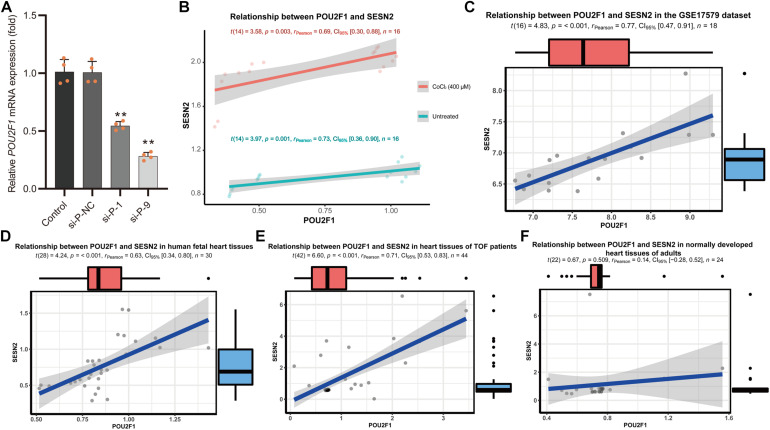
Positive relationship between *POU2F1* expression levels and *SESN2* expression levels. **(A)** RNA interference targeting *POU2F1* (si-P-1; 5′-GCAGGCUUUCCUUGGACAUTT-3′, and si-P-9; 5′-GGAAAUGACUUCAGCCAAATT-3′) in HEK293T cells. **P < 0.001 against negative control small interfering RNA (si-P-NC; 5′-UUCUUCGAACGUGUCACGUTT-3′). **(B)** A positive relationship between *POU2F1* expression and *SESN2* expression was found either with or without hypoxia-mimetic CoCl_2_ treatment in HEK293T cells. Red line refers to the case with 400 μM CoCl_2_ treated HEK293T cells (Pearson *r* = 0.69; *P* = 0.003) and green line refers to the case with untreated HEK293T cells (Pearson *r* = 0.73; *P* = 0.001). **(C)** Expression levels of *SESN2* were positively correlated with *POU2F1* expression levels in GSE17579 dataset (Pearson *r* = 0.77; *P* < 0.001). **(D)** Expression levels of *SESN2* were positively correlated with *POU2F1* expression levels in normal fetal heart tissues (Pearson *r* = 0.63; *P* < 0.001). The expression data were derived from GSE13834, GSE17579, GSE35776, GSE45821, GSE61154, and GSE150051. Expression profiles were normalized using R and *LIMMA* package, then the expression of β-*ACTIN* served as internal control to normalize the relative expression of *SESN2* and *POU2F1*. **(E)** Expression levels of *SESN2* were positively correlated with *POU2F1* expression levels in heart tissues of TOF patients (Pearson *r* = 0.63; *P* < 0.001). The expression data were derived from GSE14970, GSE26125, and GSE35776. Expression profiles were normalized using R and *LIMMA* package, then the expression of β-*ACTIN* served as internal control to normalize the relative expression of *SESN2* and *POU2F1*. **(F)** The significantly positive relationship between *SESN2* expression and *POU2F1* expression was not observed in normal adult heart tissues (Pearson *r* = 0.14; *P* = 0.509). The expression data were derived from GSE17579, GSE26125, GSE35776, and GSE45821. Expression profiles were normalized using R and *LIMMA* package, then the expression of β-*ACTIN* served as internal control to normalize the relative expression of *SESN2* and *POU2F1*.

The candidate SNPs rs492554, rs2481974, and rs510803 were located 12.1, 26.4, and 68.6 kb upstream of the transcription initiation start site, respectively. EMSA was performed to assess all three candidate functional variants for allelic patterns of nuclear protein binding. For rs2481974 and rs510803, alleles did not show specific or favorable binding. For rs492554, competition experiments suggested that labeled T-probe complex formation was reduced when 100× unlabeled T-probe was added, whereas a slightly weaker band was observed when 100× unlabeled C-probe was added. Similarly, the unlabeled T-probe showed effective competition with the labeled C-probe for binding factors (Figure 2B). Mass spectrometry was used to analyze proteins in the silver-stained band corresponding to the shifted band of EMSA. To assess whether the identified proteins can bind to the region around rs492554, we analyzed the DNA-binding position weight matrix data of TFs. Only human POU2F1 indicated potential binding to the DNA sequence harboring rs492554 (Figures 2C,D). Small interfering RNA (siRNA)−mediated knockdown of *POU2F1* was performed in human embryonic kidney 293T cells (Figure 3A). A positive relationship between *POU2F1* expression and *SESN2* expression was found either with or without hypoxia-mimetic treatment (Pearson *r* = 0.69; *P* = 0.003 and Pearson *r* = 0.73; *P* = 0.001, respectively) (Figure 3B). This positive correlation was also observed in the GSE17579 dataset (Pearson *r* = 0.77; *P* < 0.001) (Figure 3C), which contains three samples of undifferentiated human embryonic stem cells (hESC), three samples of hESC-derived cardiomyocytes, three samples of undifferentiated human induced pluripotent stem cells (hiPSC), three samples of hiPSC-derived cardiomyocytes, three normal fetal heart samples, and three normal adult heart samples. When considering the tissue origin, the significantly positive relationship between *POU2F1* expression and *SESN2* expression was observed in normal fetal heart tissues (Pearson *r* = 0.63; *P* < 0.001) (Figure 3D) and heart tissues of TOF patients (Pearson *r* = 0.71; *P* < 0.001) (Figure 3E), but not in normal adult heart tissues (Pearson *r* = 0.14; *P* = 0.509) (Figure 3F). These results indicate that rs492554 located in the putative enhancer element is a possible causal variant of the CHD phenotype, and POU2F1 is likely to be a driver TF involved in the regulation of rs492554 on *SESN2* expression.

### The T-C Haplotype of rs492554-rs12406992 Specifically Increases Transcriptional Activity

**FIGURE 4:**
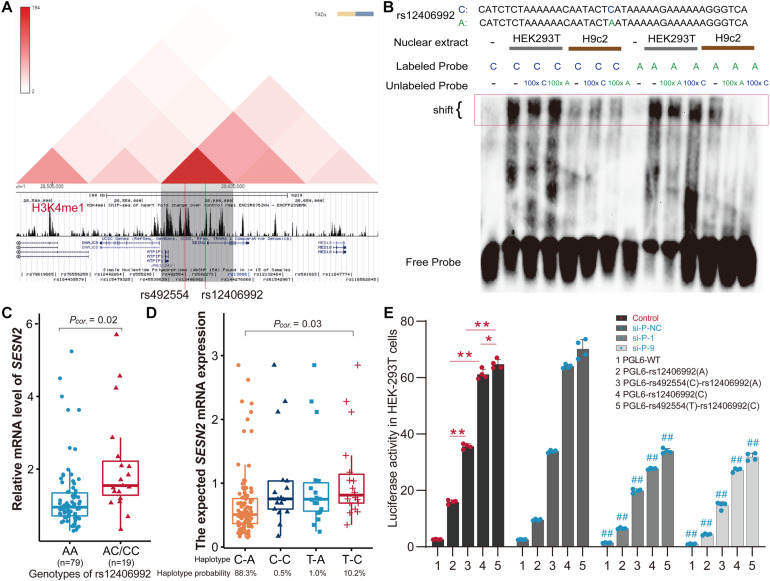
The T-C haplotype of rs492554-rs12406992 specifically increases transcriptional activity. **(A)** Hi-C plots Hi-C chromatin interaction plots of human left ventricular tissue for the region surrounding the locus of rs492554 and rs12406992. **(B)** EMSAs were performed using biotin-labeled double-stranded oligonucleotides for the C and A alleles of rs12406992 and nuclear extracts from HEK293T cells and H9c2 cells. 100× molar excess of unlabeled competitor was added in specified lanes. The rs12406992 C allele drives preferential protein binding. Blue 100× refers to the C allele-containing probe. Green 100× refers to the A allele-containing probe. **(C)** Relative mRNA expression levels of *SESN2* in 98 myocardial tissues with rs12406992 genotypes. Gene expression data were analyzed by two-tailed unpaired Student *t*-test with Welch’s correction for unequal variances. **(D)** Association between the expected *SESN2* expression and the haplotypes of rs492554-rs12406992. Expected gene expression data were analyzed by two-tailed unpaired Student *t*-test with Welch’s correction for unequal variances. Haplotype probabilities are indicated under each haplotype label. **(E)** The regions encompassing rs12406992 alleles with or without the linked rs492554 regions were cloned into the pGL6-Basic vector, and HEK293T cells were transfected. Luciferase activity was measured 48 h after transfection and was normalized against protein concentration. **P < 0.001, *P < 0.01 against empty vector pGL6-WT are shown. The luciferase activity was significantly reduced in HEK293T cells co-transfected with *POU2F1* small interfering RNA and the constructed luciferase reporter vectors. ##*P* < 0.001 against si-P-NC co-transfected with the corresponding luciferase reporter vector.

Considering that gene transcription is regulated by the interaction of *trans*-acting factors and *cis*-acting DNA elements, and distal enhancer activation is related to long-range promoter-enhancer DNA-DNA chromatin loops, we next focused on SNPs located within the annotated promoter region. The SNPs in strong or complete LD with rs492554 in the Han population (CHB and CHS) were evaluated for potential binding preference of the identified nuclear protein. Interestingly, this analysis revealed that rs12406992 (*r*^2^ = 0.845 with rs492554) was located in the putative promoter element and also mapped within the binding motif of POU2F1 (Figure 2D). The long-range interaction between the fragment carrying the rs12406992 and the fragment carrying the rs492554 was identified by a published Hi-C dataset GSE58752 from human left ventricular tissue (Figure 4A; Leung et al., 2015). Hi-C plots were visualized using the 3D Genome Browser (Wang et al., 2018).

For rs12406992, results from EMSA revealed that labeled C-probe and HEK293T cell nuclear extracts complex formation was reduced when 100× unlabeled C-probe was added, whereas a slightly weaker band was observed when 100× unlabeled A-probe was added. And the unlabeled C-probe showed effective competition with the labeled A-probe for binding factors (Figure 4B). However, this allele specific DNA binding affinity was not observed between H9c2 nuclear extract and the probes. It was similar to that observed from EMSA results of rs492554. We also detected the distribution of rs12406992 genotype in 98 myocardial tissue samples by sequencing the PCR products (Supplementary Table 6) and evaluated the correlation between rs12406992 genotype and *SESN2* expression level. The individuals carrying the rs12406992 CC or AC genotype had a higher *SESN2* expression level than AA genotype carriers (*P* = 0.02; Figure 4C). Association between *SESN2* expression and the haplotypes of rs492554-rs12406992 was further evaluated under the assumption of additive haplotype effects (Garnier et al., 2013; Brown et al., 2017). The T-C haplotype of rs492554-rs12406992 had a higher expected mean of *SESN2* expression level than C-A haplotype (*P* = 0.03; Figure 4D). Haplotype analysis of 98 samples with rs492554 and rs12406992 genotype data showed that the haplotype probabilities of C-A, T-C, T-A, and C-C were 88.3, 10.2, 1.0, and 0.5%, respectively (Figure 4D); and there was 86.9%, 11.2%, 1.0%, 1.0% in CHB; 87.6%, 10.9%, 1.0%, 0.5% in CHS. The T-C and C-A haplotypes had a haplotype probability over 5% in Chinese Han population, and were considered as further experimental haplotypes (Wang et al., 2010).

To assess POU2F1, rs12406992 and rs492554 for gene-regulatory potential, we conducted luciferase reporter assays in HEK293T cells. The rs12406992 C-allele exhibited significantly higher (3.8-fold) reporter activity than did the rs12406992 A-allele, and a significantly higher (1.8-fold) reporter activity derived from the construct containing the T-C haplotype of rs492554-rs12406992 compared to those of the construct containing the C-A haplotype (Figure 4E). The genomic region around rs492554 showed significantly higher enhancer activity for both alleles, with the protective-associated T-C haplotype exhibiting higher reporter activity. We also performed luciferase assays for reporter vectors with siRNA against *POU2F1* or control siRNA in HEK293T cells. Knockdown of *POU2F1* showed decreased transcriptional activity, and the previously observed significant allele- or haplotype-specific reporter activity was observed (Figures 3A, 4E). This allele- or haplotype-dependent transcriptional activity regulated by POU2F1 was consistent with the allele-specific expression in the eQTL data and allele-preferential binding in EMSA. Together, these data suggest that POU2F1 may play a role in *SESN2* allelic expression by binding to the rs492554 enhancer and rs12406992 promoter with preference for the CHD protective-associated T allele at rs492554 or T-C haplotype at rs492554-rs12406992.

### *Sesn2* Protects H9c2 Cells From Hypoxia-Mimetic Cobalt Chloride-Induced Cell Injury

**FIGURE 5:**
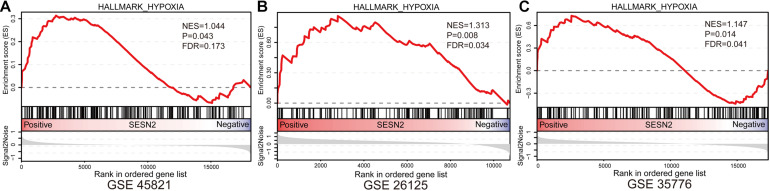
Gene Set Enrichment Analysis results of hypoxia gene set for *SESN2* high-expression samples vs. *SESN2* low-expression samples in normal fetal or adult heart tissues from **(A)** GSE45821, **(B)** GSE26125, and **(C)** GSE35776 datasets. NES, Normalization enrichment score. FDR, false discovery rate.

Given that the protective T allele at rs492554 is associated with increased levels of *SESN2* expression and confers decreased risk of CHD in high-altitude populations, we tested whether increased *SESN2* levels could lead to altered cellular phenotypes relevant to heart development. Additionally, GSEA showed that hypoxia gene set was enriched in normal fetal or adult heart tissues with high *SESN2* expression in GSE45821, GSE26125, and GSE35776 datasets from the GEO database (Figure 5). As long-term hypoxic exposure is considered to be one of the causes of heart development defects in high-altitude populations, and *SESN2* seems to be a genetic factor representing a potential link between hypoxia and CHD, we also examined whether *SESN2* is involved in regulating the phenotype of cardiomyocytes under hypoxic conditions, by overexpressing or knocking down *Sesn2* in embryonic rat-heart-derived H9c2 cells treated with or without CoCl_2_.

The concentration of CoCl_2_ was selected based on the cell viability results from our present and previous studies. First, the MTT and LDH leakage assays revealed that CoCl_2_ treatment for 5 h decreased cell viability in a concentration-dependent manner, with nearly 50% viability after 400 μM CoCl_2_ treatment (Supplementary Figures 2A,B). In addition, western blot results demonstrated that the expression of Hif1α and Sesn2 was upregulated in H9c2 cells treated with 400 μM CoCl_2_ for 5 h (Supplementary Figures 2C–E). Based on these results, a concentration of 400 μM CoCl_2_ was selected to construct the hypoxic cell model by hypoxia-mimetic CoCl_2_ in the following experiments.

**FIGURE 6:**
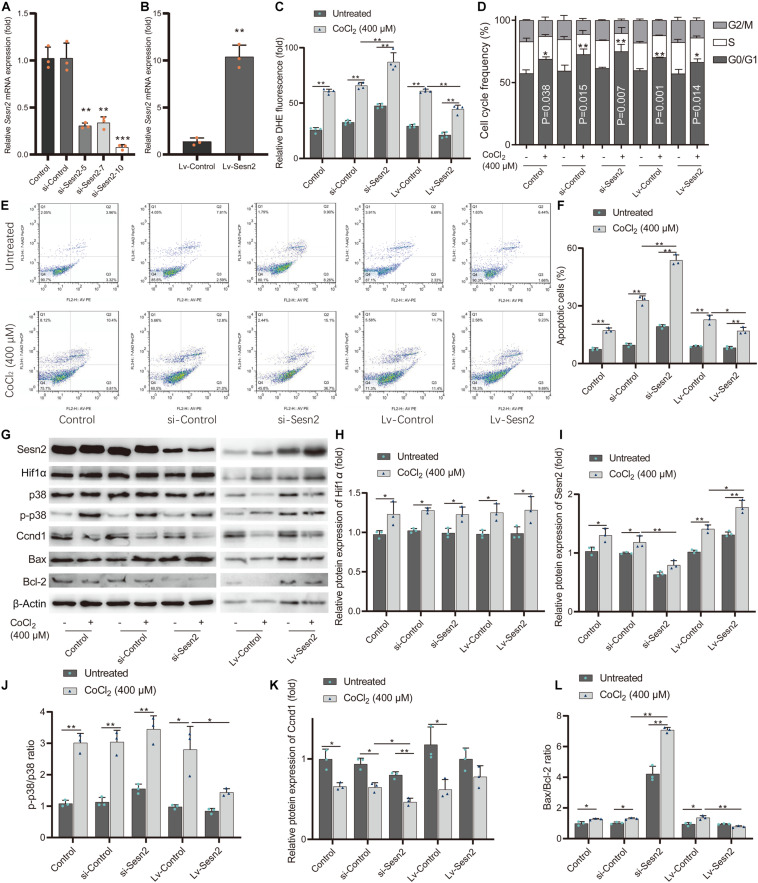
*Sesn2* protects H9c2 cells from hypoxia-mimetic cobalt chloride-induced cell injury. **(A)** *Sesn2* relative expression level in H9c2 cells after transfection with RNA interference targeting *Sesn2* (si-Sesn2-5, si-Sesn2-7, and si-Sesn2-10). **P < 0.01, ***P < 0.001 against negative control small interfering RNA (si-Control). **(B)** *Sesn2* relative expression level in H9c2 cells after transfection with empty vector (LV-Control) and overexpression vector (LV-Sesn2). **P < 0.01 against LV-Control. **(C)** Effects of knockdown or overexpression of *Sesn2* on cellular ROS with or without cobalt chloride-induced hypoxia. The DHE fluorescence intensity represents ROS concentration. **(D)** Effects of knockdown or overexpression of *Sesn2* on cell cycle distribution with or without cobalt chloride-induced hypoxia. **(E)** Apoptotic cells were quantified by Annexin V-PE/7-AAD dual staining followed by flow cytometry. **(F)** Effects of knockdown or overexpression of *Sesn2* on cell apoptosis with or without cobalt chloride-induced hypoxia. **(G)** Effects of knockdown or overexpression of *Sesn2* on the levels of Hif1α, Sesn2, p38, p-p38, Ccnd1, Bax, and Bcl-2 with or without cobalt chloride-induced hypoxia. **(H)** The relative protein expression levels of Hif1α. **(I)** The relative protein expression levels of Sesn2. **(J)** The ratio of p-p38/p38. **(K)** The relative protein expression levels of Ccnd1. **(L)** The ratio of Bax/Bcl-2. **P < 0.01, *P < 0.05. siRNA, small interfering RNA. Lv-gene, gene-overexpressed lentiviral vector.

We examined the effect of *Sesn2* on cell viability and oxidative stress by detecting LDH leakage, ROS levels, and SOD activity. First, the expression levels of *Sesn2* in *Sesn2*-knocked down and *Sesn2*-overexpressed H9c2 cells were evaluated by RT-qPCR (Figures 6A,B). The si-Sesn2-10 (5′-CAAGCAGAGACCCAUUGAATT-3′) with the highest knockdown efficiency was selected for the subsequent experiments. CoCl_2_-induced hypoxia significantly increased LDH leakage and ROS levels, and reduced SOD activity in CoCl_2_-treated H9c2 cells compared with that in control group. In addition, knockdown of *Sesn2* aggravated the CoCl_2_-induced decrease in SOD activity, and increase in LDH leakage and ROS levels, while overexpression of *Sesn2* significantly alleviated the CoCl_2_-induced decrease in SOD activity, and increase in LDH leakage and ROS levels (Figure 6C and Supplementary Figures 2F,G). We also investigated the effect of *Sesn2* on cell proliferation and apoptosis in the hypoxic cell model. Results of BrdU incorporation and cell cycle analysis showed that CoCl_2_-induced robust arrest of cell proliferation, accompanied by cell cycle arrest in the G1 phase. *Sesn2* knockdown significantly aggravated CoCl_2_-induced inhibition of cell proliferation and cell cycle G1 phase arrest observed in H9c2 cells treated with CoCl_2_ alone. Meanwhile, overexpression of *Sesn2* attenuated these observed effects (Figure 6D and Supplementary Figure 2H). Furthermore, caspase−3 activity and flow cytometry assays indicated that CoCl_2_-induced cell apoptosis was facilitated by *Sesn2* knockdown and attenuated by *Sesn2* overexpression (Figures 6E,F and Supplementary Figure 2I).

The levels of oxidative stress-related proteins phosphorylated p38 mitogen-activated protein kinase (p-p38) and p38, cell cycle-related protein Ccnd1, apoptosis-related proteins Bcl-2 and Bax were examined by western blot analysis (Figures 6G–L). Consistent with the results mentioned above, the ratio of p-p38 to p38 reflects the level of oxidative stress in each group (Figure 6J); the Ccnd1 levels reflect the level of cell cycle G1 phase arrest in each group (Figure 6K); the ratio of the pro-apoptotic protein Bax to the anti-apoptotic protein Bcl-2 also reflects the level of apoptosis in each group (Figure 6L). These results indicate that *Sesn2* protects H9c2 cells against hypoxia-mimetic CoCl_2_-induced oxidative stress and rescues hypoxia-mimetic cobalt chloride-induced cell cycle G1 phase arrest and cell apoptosis in H9c2 cells by regulating the levels of Ccnd1, Bax, and Bcl2.

## Discussion

We present a study for understanding the mechanism underlying the rs492554 CHD protective locus related to high-altitude hypoxic environment exposure from the perspective of genetic factors. Our study provides evidence that rs492554 is associated with CHD susceptibility in high-altitude populations and also has an eQTL effect on the downstream hypoxia response gene *SESN2*. *SESN2* protects against oxidative stress and cell apoptosis and maintains cell viability and proliferation in the hypoxia-mimetic cobalt chloride-induced H9c2 cell model.

Rs492554, located 12.1 kb upstream of *SESN2*, was associated with CHD in a high-altitude population, but not in a low-altitude population. However, the stability of the correlation presented by geographical differences still needs to be further evaluated in a large population. In eQTL analysis, the correlation between protective T allele and higher levels of *SESN2* expression was reproducible in myocardial tissues, including 386 left cardiac ventricular tissues from the GTEx database and 98 myocardial samples that we collected. LD analysis, functional region annotation, and EMSA revealed that rs492554 is an enhancer variant, and different alleles preferentially bind to POU2F1 to drive the expression of its eQTL gene *SESN2*. In this study, POU2F1 was only detected in nuclear extracts from HEK293T cells, but not H9c2 cells. This may be due to the different DNA binding position weight matrix between human POU2F1 and rat Pou2f1. We noted significantly positive relationship between *POU2F1* expression and *SESN2* expression in human embryonic cells, normal fetal heart tissues, and heart tissues of TOF patients, but not in normal adult heart tissues. This may be explained by the features of POU2F1 in the following. POU2F1 is a key TF in early embryonic development and is involved in the regulation of cellular oxidative stress responses (Sebastiano et al., 2010). POU2F1 has an increased affinity with *cis*-acting elements in the condition of oxidative stress-related DNA damage (Gupta and Storey, 2021).

We also noticed that rs12406992, located in the promoter region of *SESN2*, showed strong LD with rs492554 in the Han population (CHB and CHS) from the 1000 Genomes Project. Rs12406992 C allele also showed a preference for binding to POU2F1 in the DNA-binding position weight matrix motif analysis, the DNA probe containing the rs12406992 C allele had a preferential binding affinity to HEK293T cell nuclear extracts than the A allele. Although the eQTL effect of rs12406992 on *SESN2* was not found in myocardial tissues from the GTEx database, we observed that rs12406992 C allele carriers had higher levels of *SESN2* expression compared with AA carriers and the T-C haplotype of rs492554-rs12406992 had higher expected levels of *SESN2* expression compared with C-A haplotype. A potential long-range interaction between the fragment carrying the rs12406992 and the fragment carrying the rs492554 was identified from Hi-C data of human left ventricular tissue. When we evaluated the effects of different alleles of rs492554 and rs12406992 on transcriptional activity to explain the potential long-range regulation, we observed the C allele of rs12406992 and the T-C haplotype of rs492554-rs12406992 facilitated transcriptional activity, and knockdown of *POU2F1* showed significantly decreased transcriptional activity. This evidence may indicate that POU2F1 regulates the loop formation between the rs492554 enhancer and rs12406992 promoter with preferential recruitment to the C allele at rs12406992 and the T allele at rs492554. This difference in the preference of POU2F1 and rs492554, rs12406992 alleles may also be related to other co-activators, which is a possible mechanism that affects the expression of *SESN2*.

Our GSEA results showed that high expression of *SESN2* was positively correlated with hypoxia gene set in normal fetal or adult heart tissues. Exposure to high-altitude hypoxic environments is considered to be one of the causes of the high incidence of CHD in high-altitude populations (Bae et al., 2003; Zhang, 2005; Patterson and Zhang, 2010; Chun et al., 2019; Li J. J. et al., 2019). These data imply that *SESN2* functionally mediates the CHD risk associated with exposure to a hypoxic environment at rs492554. Therefore, we used the hypoxia-mimetic CoCl_2_-induced H9c2 cell model to perform preliminary explorations and describe the regulatory mechanism of this association, in order to provide more clues for CHD risk assessment. Here, we observed that chemical hypoxia caused by cobalt chloride increased Hif1α and Sesn2 expression in H9c2 cells. The mechanism for chemical hypoxia-induced injury included excessive oxidative stress, cell proliferation arrest, and cell apoptosis accompanied by increased LDH leakage, ROS levels, p-p38 levels, caspase−3 activity, and the ratio of Bax to Bcl-2, and decreased SOD activity and Ccnd1 expression. Furthermore, chemical hypoxia-induced injury was significantly aggravated by *Sesn2* knockdown, but partially reversed by *Sesn2* overexpression with the corresponding molecular mechanisms. These results indicate that *Sesn2* protects against chemical hypoxia-induced H9c2 cell injury by decreasing oxidative stress and cell apoptosis and maintains cell viability and cell proliferation. Similarly, previous studies reported that *SESN2* exerts a cardioprotective effect against damage caused by lipopolysaccharide, doxorubicin, and aging (Hwang et al., 2018; Li R. et al., 2019; Liu et al., 2020). This also emphasizes the important role of *SESN2* in heart development. Collectively, these data may shed some light on one possible molecular mechanism by which the protective T allele of rs492554 protects against CHD by increasing *SESN2* expression. However, our research on the mechanism in H9c2 cells is limited. POU2F1 cannot directly and effectively evaluate the expression level of *SESN2* in different genotypes under oxidative stress. Further studies are required to establish the detailed causal link.

The present study shows that the rs492554 protective T allele enhances the chromatin binding of POU2F1 and elevates *SESN2* expression, thereby ameliorating possible cell injury caused by hypoxic exposure and reducing CHD risk in high-altitude populations. POU2F1 may mediate long-range enhancer-promoter interactions by preferentially binding to the rs492554-T allele and rs12406992-C allele. However, the genetic regulatory mechanism involved in the eQTL effect of rs492554 on *SESN2* in myocardial tissues and the biological function of *SESN2* during cardiac development under hypoxic stress needs further clarification. Notably, an association between rs492554 and CHD risk was observed in the high-altitude but not in the low-altitude Han Chinese population. The interaction between *SESN2* rs492554 polymorphism and high-altitude hypoxic environments may be one of the reasons for the high incidence of CHD at high-altitude. Considering the insufficient statistical power caused by limited sample size, well-designed studies with large sample sizes and different ethnic and geographical populations are needed to test the reliability of rs492554 as a CHD predictive marker.

## Data Availability Statement

The datasets analyzed for this study can be found in the GEO datasets (GSE13834, GSE14970, GSE17579, GSE26125, GSE35776, GSE45821, GSE61154, GSE150051, and GSE58752) and 1000 Genomes Project (http://asia.ensembl.org/Homo_sapiens/Info/Index). The datasets generated for this study are included in the article/Supplementary Material.

## Ethics Statement

This study was approved by the Ethics Committees of Lanzhou University of Basic Medical Science (20160204). Written informed consent to participate in this study was provided by the participants’ legal guardian/next of kin.

## Author Contributions

XX, YL, and WY designed the study. XX obtained financial support. XX and WY conducted the bioinformatics analyses. WY, JB, TY, KY, and DX enrolled the samples and clinical data. WY, YL, and JB performed the experiments and participated in data analysis. WY and YL drafted the manuscript. XX, DX, and XZ revised the manuscript critically. All authors read and approved the final manuscript.

## Conflict of Interest

The authors declare that the research was conducted in the absence of any commercial or financial relationships that could be construed as a potential conflict of interest.

## Footnotes

1 http://www.gtexportal.org/

2 http://www.ncbi.nlm.nih.gov/geo/
