# Supplementary material for: A Functional Variant Rs492554 Associated With Congenital Heart Defects Modulates SESN2 Expression Through POU2F1
Source: Front Cell Dev Biol. 2021 Jun 23;9:668474. doi: 10.3389/fcell.2021.668474 (PMC8260953; doi:10.3389/fcell.2021.668474)
Supplement: Supplementary file 1 [file Data_Sheet_1.docx]

Supplementary Material

**A Functional Rs492554 Variant Associated with Congenital Heart Defects Modulates *SESN2* Expression through POU2F1**

**Supplementary Table 1**. List of potential CHD-related dominant genotypes identified by exome sequencing from three families in high-altitude areas. See Supplemental Excel file (Supplementary Table 1).

**Supplementary Table 2**. Primer sequences for PCR or RT-qPCR

| Species | SNP/Gene | Primer sequences (5’-3’) | |
| --- | --- | --- | --- |
| Human | rs12406992 (C) | Forward | AAACAATACTCATAAAAAGAAAAAAGGGTCA |
|  |  | Reverse | TTCTTTTTATGAGTATTGTTTTTTAGAGATG |
|  | rs2274848 | Forward | CCACCCACTGCTTGTTCGTA |
|  |  | Reverse | GGCCCTCTATTCTGCCTTCC |
|  | rs492554 | Forward | ATCAGGACCCGGTTGATAGG |
|  |  | Reverse | GCTCCTCTTACGTAAACCGCA |
|  | rs12406992 | Forward | GGCTGAGGAGGGAATATCGC |
|  |  | Reverse | CCAAGTCCCCCTCTGAGACT |
|  | *SESN2* | Forward | CCAGAGAAGACCACCCGAAG |
|  |  | Reverse | CAGGTCATGTAGCGGGTGAT |
|  | *POU2F1* | Forward | CAACACAGGCACACAAACCAA |
|  |  | Reverse | GCCTGTAAACTTGTTCCAGCG |
|  | *β-ACTIN* | Forward | GAAACTACCTTCAACTCCATC |
|  |  | Reverse | CTAGAAGCATTTGCGGTGGAC |
| Rat | *Sesn2* | Forward | TCAGCGGATATTCTGGAGCC |
|  |  | Reverse | ATGGAGGTGTCTACGCCACT |
|  | *β-Actin* | Forward | CCACCATGTACCCAGGCATT |
|  |  | Reverse | GAGCCACCAATCCACACAGA |

**Supplementary Table 3**. Western blot antibodies used in this study

| Antibody | Company | Catalog number | Application |
| --- | --- | --- | --- |
| p38 | Cell Signaling | 9212 | 1:1000 |
| p-p38 | Cell Signaling | 9211 | 1:1000 |
| Hif1α | Abcam | Ab179483 | 1:1000 |
| Sesn2 | Abcam | ab178518 | 1:1000 |
| Ccnd1 | Abcam | ab16663 | 1:100 |
| Bax | Proteintech | 50599-2-Ig | 1:5000 |
| Bcl-2 | Proteintech | 26593-1-AP | 1:1000 |
| β-Actin | Proteintech | 20536-1-AP | 1:2000 |
| Goat Anti-Rabbit IgG(H+L) | Proteintech | SA00001-2 | 1:2000 |

**Supplementary Table 4**. Association of rs2274848 polymorphism and CHD in two independent case-control groups.

| Variable | rs2274848 Genotype | Case | Control | OR (95% CI) | p Value |
| --- | --- | --- | --- | --- | --- |
| Beijing group |  |  |  |  |  |
|  | G vs. A | 11/229 | 14/246 | 0.84 (0.38-1.90) | 0.84 |
|  | AG vs. AA | 11/109 | 12/117 | 0.98 (0.42-2.30) | 0.99 |
|  | GG vs. AA | 0/109 | 1/117 | 0.36 (0.01-8.90) | 1.00 |
|  | AG+GG vs. AA | 11/109 | 13/117 | 0.91 (0.39-2.10) | 0.83 |
| Gansu group |  |  |  |  |  |
|  | G vs. A | 77/1239 | 100/1262 | 0.78 (0.58-1.10) | 0.14 |
|  | AG vs. AA | 73/583 | 92/585 | 0.80 (0.57-1.10) | 0.18 |
|  | GG vs. AA | 2/583 | 4/585 | 0.50 (0.09-2.70) | 0.69 |
|  | AG+GG vs. AA | 75/583 | 96/585 | 0.78 (0.57-1.10) | 0.14 |

**Supplementary Table 5**. Stratified analysis of rs2274848 by CHD subtypes in the dominant model (AG+GG vs. AA)

|  | | Beijing group | | | | Gansu group | | | | Combined group | | | |
| --- | --- | --- | --- | --- | --- | --- | --- | --- | --- | --- | --- | --- | --- |
|  | | *P* | | OR (95% CI) | | *P* | | OR (95% CI) | | *P* | | OR (95% CI) | |
| CHD classification^a^ | | |  | |  | |  | |  | |  | |  |
| Isolated CHD | 0.99 | | 0.87 (0.29-2.60) | | 0.17 | | 0.77 (0.53-1.10) | | 0.19 | | 0.79 (0.55-1.10) | |  |
| Complex CHD | 0.99 | | 0.95 (0.34-2.60) | | 0.32 | | 0.78 (0.50-1.20) | | 0.31 | | 0.80 (0.53-1.20) | |  |
| Detailed CHD phenotypes | | | | |  | |  | |  | |  | |  |
| ASD | 0.99 | | 0.75 (0.09-6.20) | | 0.17 | | 0.66 (0.38-1.10) | | 0.18 | | 0.69 (0.41-1.20) | |  |
| VSD | 0.99 | | 0.90 (0.28-2.90) | | 0.52 | | 0.84 (0.54-1.30) | | 0.48 | | 0.85 (0.57-1.30) | |  |
| TOF | 0.99 | | 0.50 (0.06-4.10) | | 0.57 | | 0.74 (0.33-1.70) | | 0.40 | | 0.68 (0.32-1.40) | |  |

^a^Isolated CHD including ASD and VSD. Complex CHD including TOF, ASD+VSD, AVSD, TOF + ASD, ASD + PDA, VSD + PDA, ASD + PS, and VSD + PS.

CHD, congenital heart disease; ASD, atrial septal defect; VSD, ventricular septal defect; TOF, tetralogy of Fallot; AVSD, atrioventricular septal defect; PDA, patent ductus arteriosus; PS, pulmonary stenosis.

**Supplementary Table 6**. The distribution of rs492554 and rs12406992 genotypes in 98 myocardial tissue samples. See Supplemental Excel file (Supplementary Table 6).


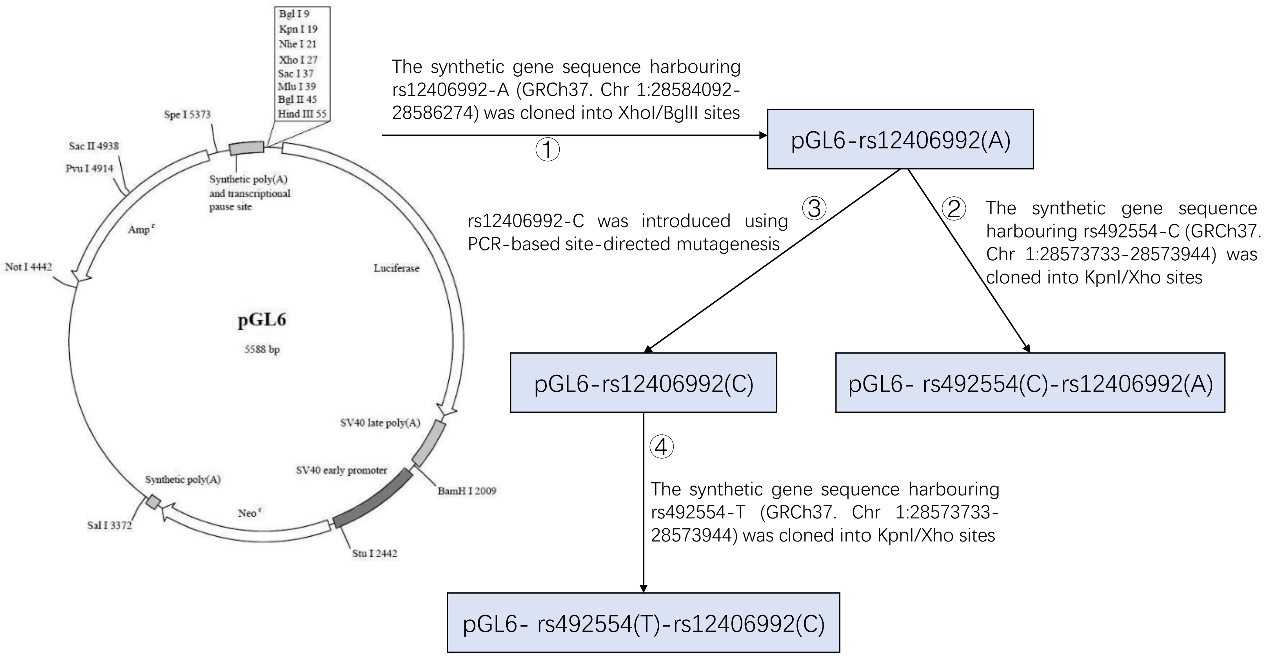


**Supplementary Figure 1** A schematic diagram of the construction of the luciferase reporter gene vectors.


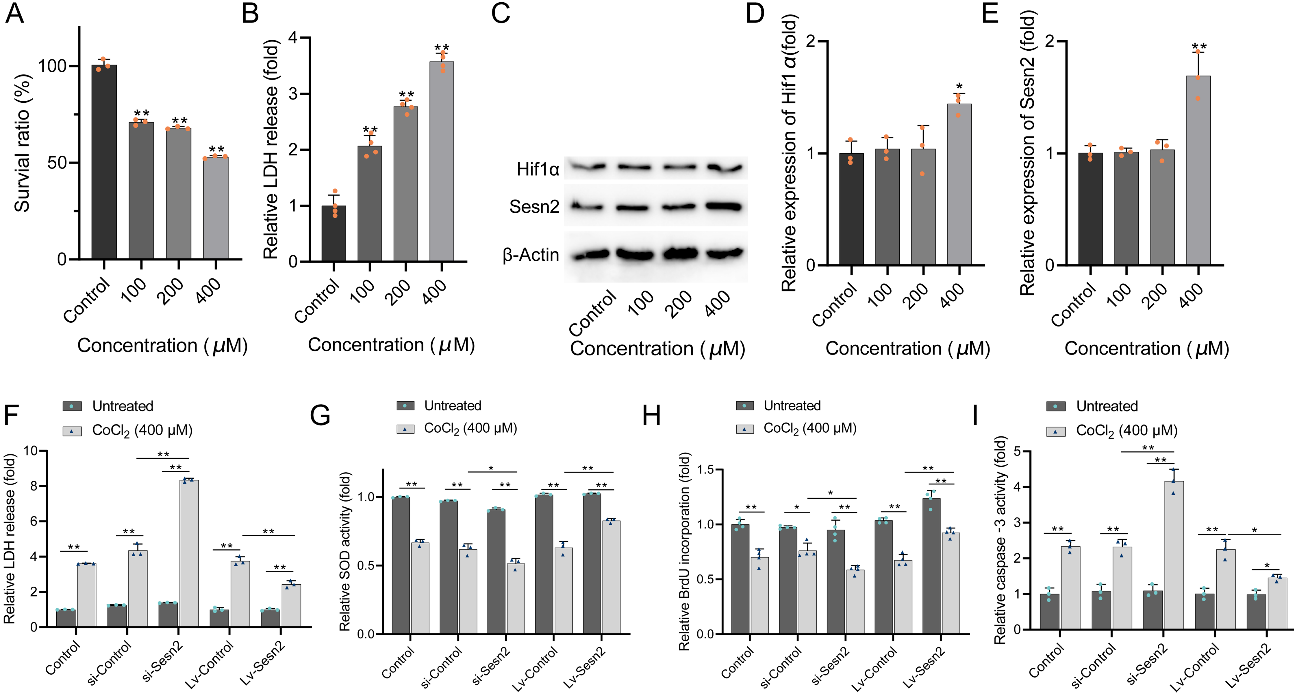


**Supplementary Figure 2** *Sesn2* protects H9c2 cells from hypoxia-mimetic cobalt chloride-induced cell injury.

**(A)** Effect of different concentrations of cobalt chloride on cell viability by MTT assay.

**(B)** Effect of different concentrations of cobalt chloride on LDH release by LDH leakage assay.

**(C)** Effect of different concentrations of cobalt chloride on the expression levels of Hif1α and Sesn2 were determined by Western blotting.

**(D)** The relative protein expression levels of Hif1α.

**(E)** The relative protein expression levels of Sesn2.

**(F)** Effects of knockdown or overexpression of *Sesn2* on LDH release with or without cobalt chloride-induced hypoxia.

**(G)** Effects of knockdown or overexpression of *Sesn2* on SOD activity with or without cobalt chloride-induced hypoxia.

**(H)** Effects of knockdown or overexpression of *Sesn2* on BrdU incorporation with or without cobalt chloride-induced hypoxia.

**(I)** Effects of knockdown or overexpression of *Sesn2* on caspase‐3 activity with or without cobalt chloride-induced hypoxia.

**P<0.01, *P<0.05. siRNA, small interfering RNA. Lv-gene, gene-overexpressed lentiviral vector.
